# Starch-Capped AgNPs’ as Potential Cytotoxic Agents against Prostate Cancer Cells

**DOI:** 10.3390/nano11020256

**Published:** 2021-01-20

**Authors:** Mariana Morais, Vera Machado, Francisca Dias, Carlos Palmeira, Gabriela Martins, Magda Fonseca, Catarina S. M. Martins, Ana Luísa Teixeira, João A. V. Prior, Rui Medeiros

**Affiliations:** 1 Molecular Oncology and Viral Pathology Group, IPO-Porto Research Center (CI-IPOP), Portuguese Oncology Institute of Porto (IPO-Porto), Research Center-LAB2, E Bdg 1st floor, Rua Dr António Bernardino de Almeida, 4200-072 Porto, Portugal; mariana.gomes.morais@ipoporto.min-saude.pt (M.M.); vera.pereira.machado@ipoporto.min-saude.pt (V.M.); francisca.carvalho.dias@ipoporto.min-saude.pt (F.D.); 2 Research Department, LPCC-Portuguese League Against Cancer (NRNorte), Estrada Interior da Circunvalação 6657, 4200-172 Porto, Portugal; 3 ICBAS, Abel Salazar Institute for the Biomedical Sciences, University of Porto, 4050-513 Porto, Portugal; 4 Department of Immunology, Portuguese Oncology Institute of Porto (IPO-Porto), Rua Dr António Bernardino de Almeida, 4200-072 Porto, Portugal; carlospalmeira@ipoporto.min-saude.pt (C.P.); gmartins@ipoporto.min-saude.pt (G.M.); 5 Experimental Pathology and Therapeutics Group, IPO-Porto Research Center (CI-IPOP), Portuguese Oncology Institute of Porto (IPO-Porto), Research Center-LAB2, E Bdg 1st floor, Rua Dr António Bernardino de Almeida, 4200-072 Porto, Portugal; 6 Biomedical Research Center (CEBIMED), Faculty of Health Sciences of Fernando Pessoa University (UFP), Praça 9 de Abril 349, 4249-004 Porto, Portugal; 7 LAQV, REQUIMTE, Laboratory of Applied Chemistry, Department of Chemical Sciences, Faculty of Pharmacy, University of Porto, 4050-313 Porto, Portugal; magdapcfonseca81@gmail.com (M.F.); catsofiamartins@gmail.com (C.S.M.M.); 8 Faculty of Medicine, University of Porto (FMUP), Alameda Prof. Hernâni Monteiro, 4200-319 Porto, Portugal

**Keywords:** silver nanoparticles, prostate cancer, green synthesis, starch, cytotoxicity, anticancer agents

## Abstract

One of the major therapeutic approaches of prostate cancer (PC) is androgen deprivation therapy (ADT), but patients develop resistance within 2–3 years, making the development of new therapeutic approaches of great importance. Silver nanoparticles (AgNPs) synthesized through green approaches have been studied as anticancer agents because of their physical-chemical properties. This study explored the cytotoxic capacity of starch-capped AgNPs, synthesized through green methods, in LNCaP and in PC-3 cells, a hormonal-sensitive and hormone-resistant PC cell line, respectively. These AgNPs were synthesized in a microwave pressurized synthesizer and characterized by ultraviolet-visible (UV-Vis) spectroscopy, transmission electron microscopy (TEM), and energy-dispersive X-ray spectroscopy (EDX). Their cytotoxicity was assessed regarding their ability to alter morphological aspect (optical microscopy), induce damage in cytoplasmic membrane (Trypan Blue Assay), mitochondria (WST-1 assay), cellular proliferation (BrdU assay), and cell cycle (Propidium iodide and flow-cytometry). AgNPs showed surface plasmon resonance (SPR) of approximately 408 nm and average size of 3 nm. The starch-capped AgNPs successfully induced damage in cytoplasmic membrane and mitochondria, at concentrations equal and above 20 ppm. These damages lead to cell cycle arrest in G0/G1 and G2/M, blockage of proliferation and death in LNCaP and PC-3 cells, respectively. This data shows these AgNPs’ potential as anticancer agents for the different stages of PC.

## 1. Introduction

Cancer is a major health problem worldwide with approximately 18.1 million new cancer cases and 9.6 million cancer deaths registered worldwide in 2018. Moreover, it is estimated that this incidence rate will double by 2035, implying an increase of the social and economic burden in the society [1].

Prostate cancer (PC) is the second most frequent cancer diagnosed in men and the fifth leading cause of death worldwide [1,2]. At the time of diagnosis, most men present localized disease, with only 5 to 10% of PC patients presenting metastatic disease. However, patients frequently recur after local treatment [3]. In fact, while five-year survival is almost 100% for patients with local and locally advanced disease, it drastically decreases for less than 30% in patients with metastatic disease [3]. Since these tumors are highly dependent of the androgen pathway, one of the major therapeutic approaches applied in advanced/metastatic PC is the androgen deprivation therapy (ADT) [4]. The Androgen receptor (AR) is a nuclear ligand transcription factor that is part of the steroid hormone nuclear receptor family and whose main ligands are the male sexual hormones, such as testosterone and dihydrotestosterone (DHT) [5]. Through ADT, the androgens levels are downregulated and consequently the cell proliferation decreases, and the apoptosis rate increases [6]. Despite the initial high response rates (from 80 to 90%) nearly all men develop resistance within 2 to 3 years, progressing to castration resistant PC (CRPC) [3]. In the last few years, new drugs with increased sensitivity or aiming to new targets have come up. However, they also showed limited benefits leading to patients’ relapse [7]. Thus, overcoming treatment resistance remains the major challenge in PC patients’ management.

In the last few years, silver nanoparticles (AgNPs) have been pointed out as potential therapeutic agents in cancer treatment, because of their specific chemical and physical properties [8]. Nanoparticles are small organized structures ranging in size between 1–100 nm [9]. Among them, AgNPs have specific physicochemical properties that grant their application in several biomedical fields, like cancer treatment [10]. Their synthesis through green approaches is very appealing because they use benign and renewable materials, are less expensive and faster, with single-step procedures and show better biocompatibility with healthy cells [11]. Their effects are highly dependent on their shape, size, and composition, as well as on the cell characteristics, suggesting a specific-cell type effect [12]. In fact, AgNPs with a spherical shape, when smaller than 100 nm, are uptaken more by cells. Also, smaller nanoparticles are uptaken more than larger ones and will interact more with the different biomolecules inside the cells. Moreover, the effect of AgNPs highly varies depending on the capping composition, even for the same cell line [13]. 

In this study, we intended to explore the cytotoxic capacity of AgNPs, synthesized through green methods, in PC management. Considering the Warburg effect that states the increased glucose consumption by cancer cells and which is already described in the more advanced stages of PC, AgNPs were synthesized using glucose as reducing agent and starch as carbohydrates-based capping and core stabilizer in order to favor their uptake by the cancer cells, and to be uptaken less by normal cells [14]. The cytotoxic capacity of AgNPs will be analyzed in a hormonal sensitive PC cell line (LNCaP) and in a hormone resistant cell line (PC-3).

## 2. Materials and Methods 

### 2.1. Materials

The water used for the preparation of solutions was purified from a Milli-Q system (conductivity ≤ 0.1 µS cm^−1^) and the reagents of high analytical grade were used without any treatment process or further purification: silver nitrate (LabKem, Barcelona, Spain), D-glucose anhydrous (Fisher Chemical, Leicestershire, U.K.) and potato starch soluble (Merck, Darmstadt, Germany).

Roswell Park Memorial Institute-1640 medium (RPMI-1640), fetal bovine serum (FBS), penicillin-streptomycin and trypsin-EDTA were purchased from Gibco^®^ (Gaithersburg, MD, USA). Trypan Blue, which allows for the evaluation of cytoplasmic membrane damage, was purchased from VWR^TM^ (Radnor, PA, USA); WST-1, which allows for the evaluation of mitochondrial capacity, was purchased from abcam^®^ (Cambridge, UK). BrdU, which allows for the evaluation of cell proliferation capacity, was purchased from ROCHE^®^ (Basel, Switzerland). Propidium iodide (PI), which allows for the evaluation of cell cycle, was purchased from Sigma-Aldrich^®^ (St. Louis, MO, USA).

### 2.2. Synthesis and Characterization of AgNPs

To synthesize the starch capped AgNPs, 5.000 mL of a glucose solution of 0.06 mol·L^−1^, 5.000 mL of AgNO3 0.04 mol·L^−1^ and 4.250 mL of potato starch solution (0.8% m/v) were mixed in a vial. The vial was placed in a microwave pressurized synthesizer (CEM^®^ Discover SP, 2.5 GHz MW, 300 W, Matthews, North Carolina, USA) under the following conditions: 150 °C, 270 s and high stirring. The synthesized AgNPs were characterized using ultraviolet-visible (UV-Vis) spectroscopy (Jasco, model V-660, Easton, MD, USA), transmission electron microscopy (TEM) and energy-dispersive X-ray spectroscopy (EDX). The UV-Vis spectra between 300 and 700 nm was recorded using a Jasco V-660 spectrophotometer. The TEM and EDX analysis were performed using the electron microscope JEOL 2100 (JEOL Ltd., Tokyo, Japan), operating at 200 kV with a high brightness LaB6 electron gun, equipped with a fast-readout “OneView” 4 k × 4 k CCD camera and an energy dispersive X-ray spectrometer from Oxford Instruments with a solid angle of 0.13 sr. For this, the solution was previously shaken, then was dropped onto a 400 mesh carbon on copper grid from Ted Pella and the solution was dried over filter paper. Considering the number of cellular assays executed throughout the present work, it was necessary to prepare several suspensions of nanoparticles to guarantee the use of freshly prepared suspensions. The concentrations of the suspensions used in this work were the total silver content obtained by atomic absorption spectrometry (AAS), as already explored in other works which used non-purified suspensions [10,15,16,17]. The silver content in the nanoparticles suspensions was determined through flame atomic absorption spectrometry (AAS) in a Perkin Elmer AAnalyst 200 spectrometer.

### 2.3. Cell Culture and Treatments Conditions

PC-3 and LNCaP cells (ATCC^®^ CRL-1435™ RRID:CVCL_0035 and ATCC^®^ CRL-1740™ RRID:CVCL_1379, respectively), obtained from ATCC (Manassas, Virginia, USA) were cultured in RPMI-1640 medium supplemented with 10% fetal bovine serum (FBS) and 1% penicillin-streptomycin and were maintained in a humidified incubator at 37 °C, 5% CO_2_, and 95% humidity. Cells were routinely grown in plastic tissue culture flasks and harvested with a solution of trypsin-EDTA, while in a logarithmic growth phase. All cells were routinely tested, each two weeks, for mycoplasma presence and were found to be free from contamination. Cells were used between passage numbers 16 and 23.

Cells were cultured in 6-multi-well plates at a density of 2 × 105 cells per mL and treated with increasing concentrations of AgNPs, ranging from 5 to 210 ppm for 24 h and 48 h. The influence of starch alone was evaluated. Incubation of cells with RPMI-1640 culture medium alone was used as negative control.

### 2.4. Trypan Blue Exclusion Assay

A trypan blue exclusion assay was performed according to the method described by Warren Strober [18]. After treatment with AgNPs, cells were trypsinized for 4 min at 37 °C, then neutralized with growth media and centrifuged at 1200 rpm for 3 min. The cell pellets were resuspended in growth media and 10 µL of cell suspension was added to 10 µL of trypan blue solution (VWR^TM^) in order to determine live cell numbers. The cells were counted in an automatic cell counter (EVE^TM^-NanoEntek, VWR^TM^) and results were expressed as percentage of viability related to control cells. Each condition was performed in triplicated.

### 2.5. Cell Morphology 

PC-3 and LNCaP cells were plated in 6-multi-well plates and the cell morphology was analyzed at 24 h and 48 h posttreatment. The cells were examined with an OLYMPUS IX51 microscope (Tokyo, Japan) using the appropriate filter sets, to identify the morphological changes compared with the untreated cells. Each condition was performed in triplicated.

### 2.6. WST-1 Viability Assay

Cellular viability was quantified in 96-well culture plates using the cell proliferation reagent WST-1 (Abcam, Cambridge, UK). At each time point, WST-1 reagent (10 µL) was added into each well and cells were incubated for an additional 2 h. Absorbance was measured with a spectrophotometer (FLUOstar Omega, BMG Labtech, Ortenberg Germany) at 450 nm with 650 nm as reference wavelength and was directly proportional to the viability of the cells. Each condition was performed eight times. The optical density (OD) value was used to calculate the percentage of cell viability by using the following formula:

(1) $$Percentage\;of\;cell\;viability=\frac{OD\;value\;of\;experimental\;AgNPs\;treated\;sample}{OD\;value\;of\;experimental\;untreated\;sample}\times 100$$

### 2.7. Cell Proliferation Assay-BrdU

The cells were grown overnight in 96-well microplates and incubated with AgNPs at 10 to 210 ppm or control (vehicle alone) for 24 h and 48 h. Cells were also incubated with 5′-bromodeoxyuridine (BrdU) solution at a final concentration of 0.01 mM during the treatment period. Optical density of proliferating cells (positive for BrdU) after ELISA assay using anti-BrdU specific antibodies (Roche Diagnostics, Mannheim, Germany) was evaluated at the microplate reader according to the manufacturer’s instructions. Results were expressed as percentage of control (100%). Each condition was performed eight times.

### 2.8. Cell Cycle Analysis

Cell cycle analysis was performed by measuring the DNA content of the treated and untreated cells using Propidium Iodide (PI) by flow cytometry. PC-3 cells were grown in 6-well plates (2 wells per condition) and incubated with AgNPs as reported in the section entitled “Cell culture and treatment conditions”. At each time point, culture medium was collected, and the cells detached with trypsin/EDTA and centrifuged at 1200 rpm for 3 min. The pellet was washed with PBS and fixed by slowly adding 3 mL of cold 70% (*v*/*v*) ethanol for 15 min at 4 °C. Fixed cells were centrifuged and the pellet obtained was washed with cold PBS. Subsequently, the cells were stained with a solution containing Triton X-100 (1.1%, *v*/*v*), PI and RNase A (50 μg/mL and 100 μg/mL, respectively) in PBS. Analysis was performed using a flow cytometer (Cytomics FC500, Beckman Coulter Brea, Califórnia, EUA). The percentage of cells in the sub-G1 phase of the cell cycle was calculated from the total 10,000 cells (100%) in the assay, and that for cells in G0/G1, S and G2/M phases was calculated from the total cells excluding the sub-G1 cells. Each condition was performed in triplicated.

### 2.9. AgNPs Uptake Measurement

To infer the AgNPs uptake by LNCaP and PC-3 cells, these cells were cultured in 6-multi-well plates at a density of 2 × 10^5^ cells per mL and treated with increasing concentrations of AgNPs, ranging from 5 to 100 ppm for 24 h and 48 h. After treatment with AgNPs, cells were trypsinized for 4 min at 37 °C, then neutralized with growth media and centrifuged at 1200 rpm for 3 min. The cells’ pellet was washed with PBS and then resuspended in NHO_3_ (5% *v*/*v*) solution and its silver content was determined through flame atomic absorption spectrometry (AAS).

### 2.10. Statistical Analysis

Using the sample T, the Mann-Whitney and the Kruskal Wallis test (depending whether the results followed or not a normal distribution), the results were analyzed via SPSS26 software (release 26, SPSS Inc., Chicago, IL, USA). *p*-Values less than <0.05 were regarded as statistically significant while all assays were repeated at least three times.

## 3. Results

### 3.1. AgNPs Synthesis and Characterization

**Figure 1:**
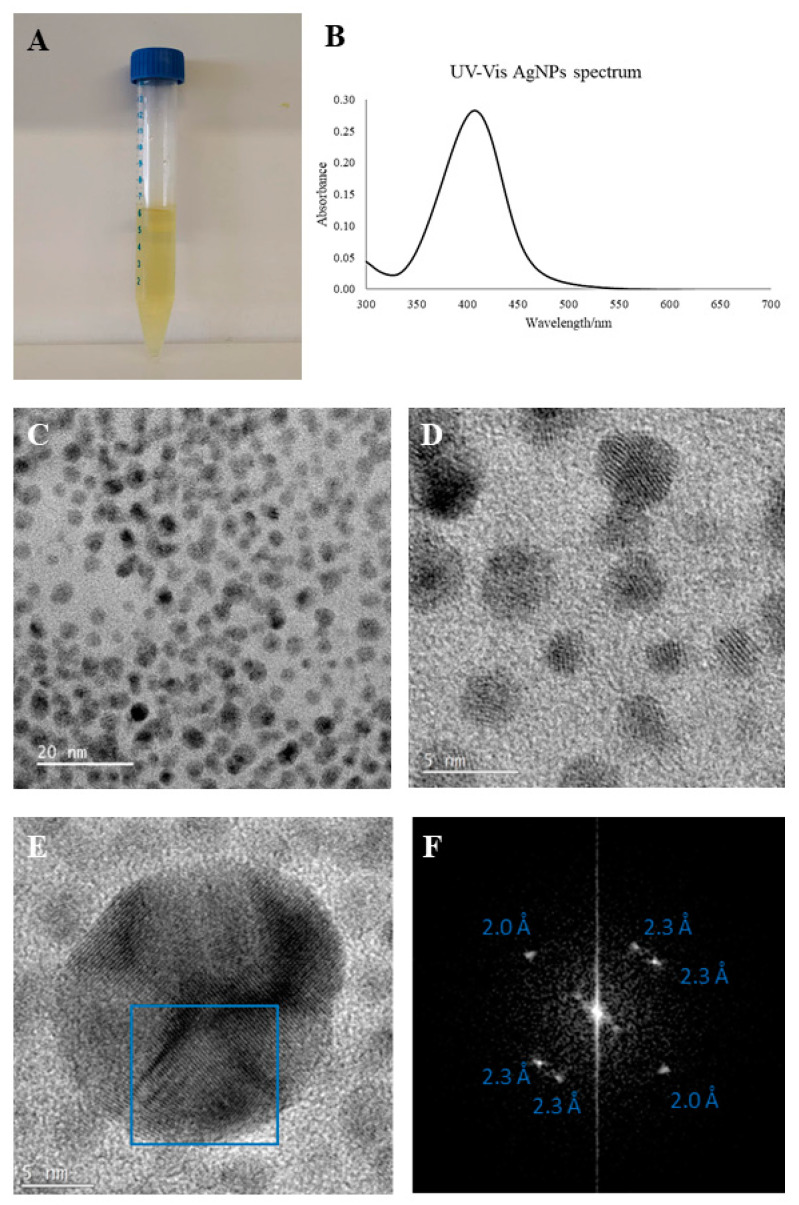
(**A**) Photograph of starch capped AgNPs’ suspension; (**B**) UV-Vis spectrum of starch capped AgNPs’ suspension; (**C**) TEM image of AgNPs (20 nm resolution); (**D**) TEM image of AgNPs (5 nm resolution); (**E**) HRTEM image of AgNPs; (**F**) FFT from the blue box in Figure 1E showing spacings of ~2.3 and ~2.0 A.

The AgNPs were synthesized following a green approach using starch as capping and glucose as reducing agent, according to a procedure previously described by Kumar, S.V. et al. and adapted for this work [19]. Upon 270 s of reaction time at 150 °C in the microwave pressurized synthesizer, the mixture acquired a yellowish color typical of AgNPs colloids (Figure 1A). No sediment was observed, even after several weeks of storage (in the dark and at room temperature), indicating good stability of the nanoparticles in the suspension. The UV-Vis spectra between 300 and 700 nm showed an SPR peak with maximum at approximately 408 nm for the synthesized AgNPs (Figure 1A). The TEM observations displayed spherical, well-dispersed AgNPs with an average size of 3 nm (Figure 1B). The HRTEM shows the (111) and (200) spacing’s of silver (Figure 1C). Also, the EDX analysis showed the presence of only silver, with the additional peaks coming from the microscope’s column and the copper grid. The quantification by AAS of AgNPs suspensions revealed in average 1192 ± 157 ppm (11 ± 1, 45 mM). The tested nanoparticles concentrations were obtained by direct dilution.

### 3.2. AgNPs’ Cytotoxic Power and Impact on Cellular Morphology

**Figure 2:**
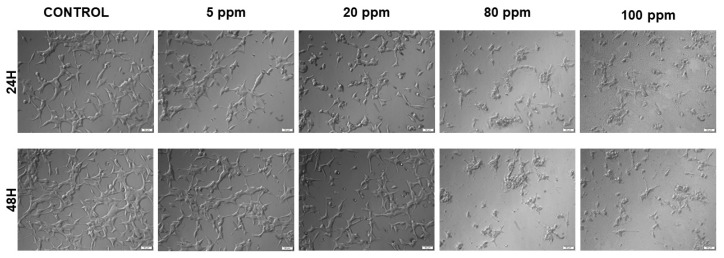
Bright field microscopy images of LNCaP cell morphology after exposure to different concentrations of AgNPs ranging from 5 to 100 ppm, for 24 h and 48 h (20× OLYMPUS IX51 microscope).

A microscopic examination of the LNCaP cell line treated with AgNPs at the different concentrations for both 24 h and 48 h is displayed in Figure 2. Both at 24 h and 48 h there is an increase of detached cells after exposure to 20 ppm of AgNPs and a significative change in cellular morphology after exposure to both 80 and 100 ppm of AgNPs.

**Figure 3:**
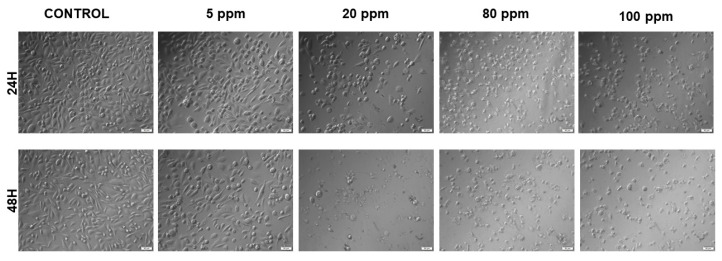
Bright field microscopy images of PC-3 cell morphology after exposure to different concentrations of AgNPs ranging from 5 to 100 ppm, for 24 h and 48 h (20× OLYMPUS IX51 microscope).

In Figure 3, one can observe the effect of the AgNPs in cellular morphology of PC-3 cells. Both at 24 and 48 h, for concentrations of 20 ppm or more it is possible to observe a shrinkage and detachment of the cells, suggesting cell death.

### 3.3. AgNPs’ Cytotoxic Power against Cytoplasmic Membrane 

The trypan blue assay is based on the principle that live cells present intact cell membranes that exclude dyes such as trypan blue, in opposition to dead cells which do not. Thus, with this assay, one can assess cell viability, especially regarding the damage of AgNPs are able to induce in the cytoplasmic membrane of cancer cells.

**Figure 4:**
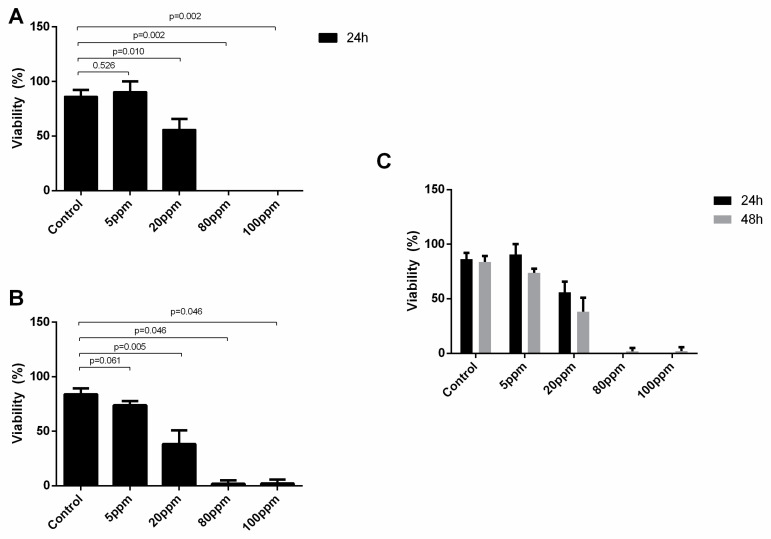
LNCaP cells viability assessed by trypan blue exclusion method upon treatment with AgNPs for 24 h (**A**) and 48 h (**B**) and the comparison between their effect at the two time points (**C**). Results are expressed as percentage of control (untreated cells), as mean ± SEM.

Regarding the LNCaP cell line, considering the two time points tested, there was no significant differences in the damage induced after 24 and 48 h of AgNPs’ exposure (Figure 4C). However, considering the AgNPs concentration, there is a significant reduction of viable cells after treatment with AgNPs at concentrations of 20 ppm (86.27 ± 5.97 vs. 56.03 ± 9.65, *p* = 0.010), 80 ppm (86.27 ± 5.97 vs. 0.00 ± 0.00, *p* = 0.002) and 100 ppm (86.27 ± 5.97 vs. 0.00 ± 0.00, *p* = 0.002) at 24 h (Figure 4A) and 48 h for 20 ppm (83.83 ± 5.45 vs. 38.13 ± 12.82, *p* = 0.005), for 80 ppm (83.83 ± 5.45 vs. 1.83 ± 3.18, *p* = 0.0046), for 100 ppm (83.83 ± 5.45 vs. 2.07 ± 3.58, *p* = 0.0046) (Figure 4B). 

**Figure 5:**
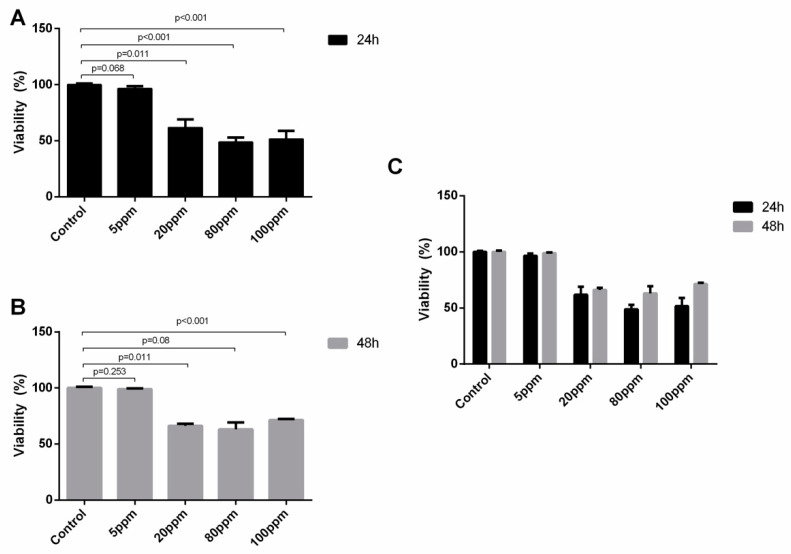
PC-3 cells viability assessed by trypan blue exclusion method upon treatment with AgNPs for 24 h (**A**) and 48 h (**B**) and the comparison between their effect at the two time points (**C**). Results are expressed as percentage of control (untreated cells), as mean ± SEM.

Regarding the PC-3 cells, considering the two time points tested, there were no significant differences in the damage induced after 24 and 48 h of AgNPs’ exposure (Figure 5C). However, considering the AgNPs concentration, there is a significant reduction of viable cells after treatment with AgNPs at concentrations of 20 ppm (100.00 ± 0.58 vs. 61.83 ± 4.16, *p* = 0.011), 80 ppm (100.00 ± 0.58 vs. 48.80 ± 2.42, *p* < 0.001) and 100 ppm (100.00 ± 0.58 vs. 51.63 ± 4.28, *p* < 0.001) at 24 h (Figure 5A) and 48 h for 20 ppm (100.00 ± 0.70 vs. 66.23 ± 1.03, *p* = 0.011), for 80 ppm (100.00 ± 0.70 vs. 63.10 ± 3.61, *p* = 0.008), for 100 ppm (100.00 ± 0.70 vs. 71.37 ± 0.61, *p* < 0.001) (Figure 5B). 

Considering the preliminary results obtained with the Trypan Blue test, one can observe that AgNPs at a concentration of 5 ppm do not show an effect in both LNCaP and PC-3 cells, at both time points. Thus, for the remaining assays, we adjusted the range of concentrations tested from 10 ppm to 210 ppm.

### 3.4. AgNPs’ Cytotoxic Power against Mitochondria

The WST-1 assay is based on the principle that tetrazolium salts are cleaved by cellular enzymes, such as mitochondrial dehydrogenases, to formazan, being an indicator of metabolic activity of cells, and thus, of their viability. Thus, with this assay, one can assess cell viability, especially regarding the damage AgNPs are able to induce in the cells’ mitochondria.

**Figure 6:**
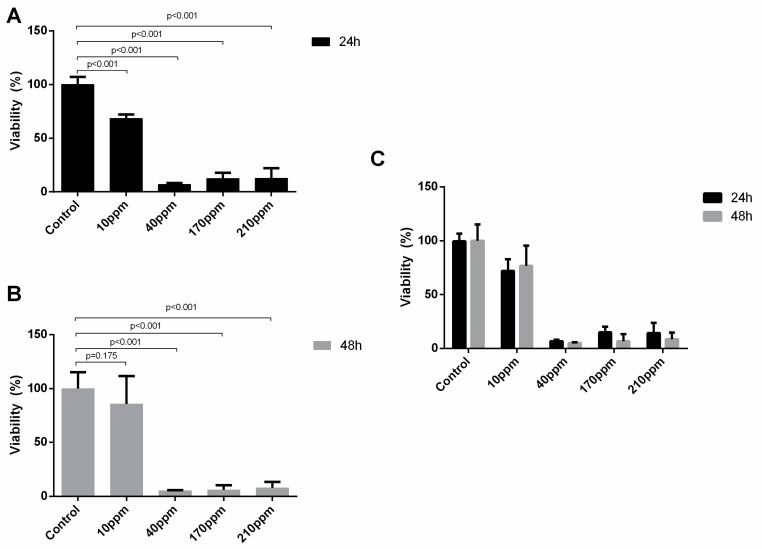
Evaluation of cell viability, by WST-1 assay, upon LNCaP cells treatment with AgNPs at concentrations of 10–210 ppm for 24 h (**A**) and 48 h (**B**) and the comparison between their effect at the two time points (**C**). Results are expressed as percentage of control (untreated cells), as mean ± SEM.

Regarding LNCaP cells, a significant reduction of viable cells after 24 h of treatment with AgNPs is observed at concentrations of 10 ppm (100.00 ± 7.16 vs. 68.30 ± 3.87, *p* < 0.001), 40 ppm (100.00 ± 7.16 vs. 6.95 ± 1.41, *p* < 0.001), 170 ppm (100.00 ± 7.16 vs. 12.42 ± 5.32, *p* < 0.001)) and 210 ppm (100.00 ± 7.16 vs. 13.18 ± 8.63, *p* < 0.001)) at 24 h (Figure 6A). After 48 h of exposure, a significant reduction of viable cells is observed at concentrations of 40 ppm (105.10 ± 9.41 vs. 5.15 ± 0.68, *p* < 0.001), 170 ppm (105.10 ± 9.41 vs. 5.97 ± 4.40, *p* < 0.001), and 210 ppm (105.10 ± 9.41 vs. 7.84 ± 5.31, *p* < 0.001),) (Figure 6B). Moreover, there was a significant reduction of viable cells after 48 h of exposure to AgNPs, when compared with 24 of exposure for 40 ppm (6.95 ± 1.41 vs. 5.14 ± 0.68, *p* = 0.018) and 170 ppm (12.42 ± 5.32 vs. 5.97 ± 4.40, *p* < 0.045) (Figure 6C). 

**Figure 7:**
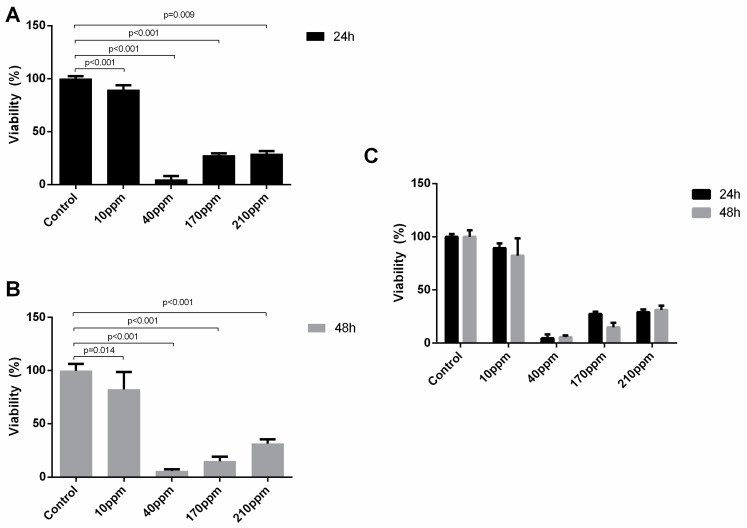
Evaluation of cell viability, by WST-1 assay, upon PC-3 cells treatment with AgNPs at concentrations of 10–210 ppm for 24 h (**A**) and 48 h (**B**) and the comparison between their effect at the two time points (**C**). Results are expressed as percentage of control (untreated cells), as mean ± SEM.

Regarding PC-3 cells, a significant reduction of viable cells after 24 h of treatment with AgNPs is observed at concentrations of 40 ppm (100.00 ± 0.89 vs. 4.60 ± 1.22, *p* < 0.001), 170 ppm (100.00 ± 0.89 vs. 27.39 ± 0.71, *p* < 0.001) and 210 ppm (100.00 ± 0.89 vs. 29.1 ± 0.89, *p* = 0.009) at 24 h (Figure 7A). This effect is kept at 48 h for AgNPs’ concentration of 40 ppm (100.00 ± 2.20 vs. 5.60 ± 0.60, *p* < 0.001), 170 ppm (100.00 ± 2.20 vs. 14.81 ± 1.53, *p* < 0.001), and 210 ppm (100.00 ± 2.20 vs. 31.37 ± 1.38, *p* < 0.001) (Figure 7B). Thus, there were no significant differences in the damage induced after 24 and 48 h of exposure to AgNPs (Figure 7C). 

### 3.5. AgNPs’ Cytotoxic Power against Cellular Proliferation

BrdU is an analog of the nucleoside thymidine. During the assay, BrdU is incorporated into replicating DNA, thus being an indicator of cellular proliferation. 

**Figure 8:**
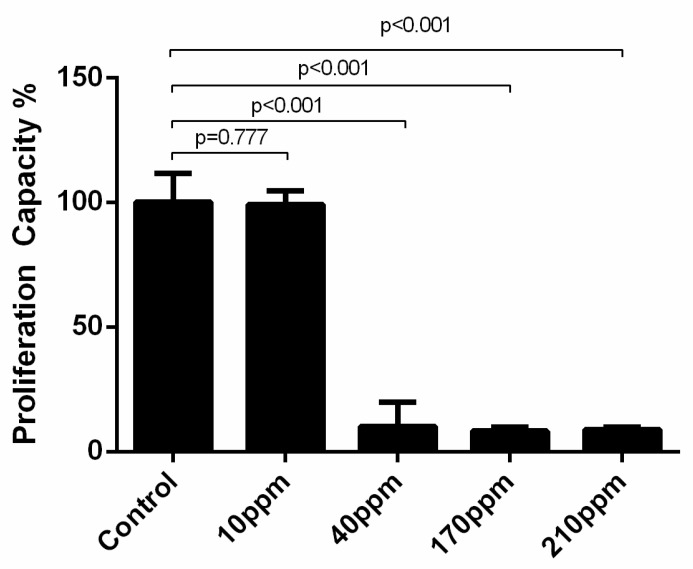
Effect of AgNPs at concentrations of 10–210 ppm, on LNCaP cell proliferation assessed by BrdU incorporation assay after 24 h. Results are expressed as percentage of control (untreated cells) considered as 100%, as mean ± SEM.

Regarding LNCaP cells, as expected, the control group cells showed a normal proliferation rate (97.60 ± 11.25). The cellular proliferation of cancer cells abruptly decreases after 24 h of treatment, when treated with AgNPs in concentrations of 40 ppm (97.60 ± 11.25 vs. 10.23 ± 9.75, *p* < 0.001), 170 ppm (97.60 ± 11.25 vs. 8.97 ± 0.49, *p* < 0.001) and 210 ppm (97.60 ± 11.25 vs. 9.28 ± 0.49, *p* < 0.001) (Figure 8).

**Figure 9:**
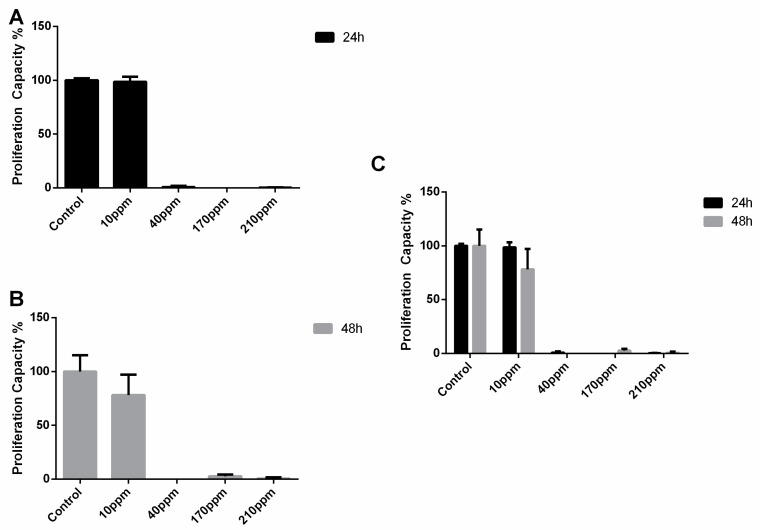
Effect of AgNPs at concentrations of 10–210 ppm, on PC-3 cell proliferation assessed by BrdU incorporation assay after 24 h (**A**) and 48 of treatment (**B**). and the comparison between their effect at the two time points (**C**). Results are expressed as percentage of control (untreated cells) considered as 100%, as mean ± SEM.

In PC-3 cells, the control group cells showed a normal proliferation rate as well. When treated with AgNPs in concentrations of 40 ppm or more, the cellular proliferation of cancer cells abruptly decreases to approximately 0% after 24 h of treatment. (Figure 9A) This effect is kept at 48 h (Figure 9B). Thus, there were no significant differences in the damage induced after 24 and 48 h of exposure to AgNPs (Figure 9C).

### 3.6. AgNPs’ Cytotoxic Power in the Cell Cycle

**Figure 10:**
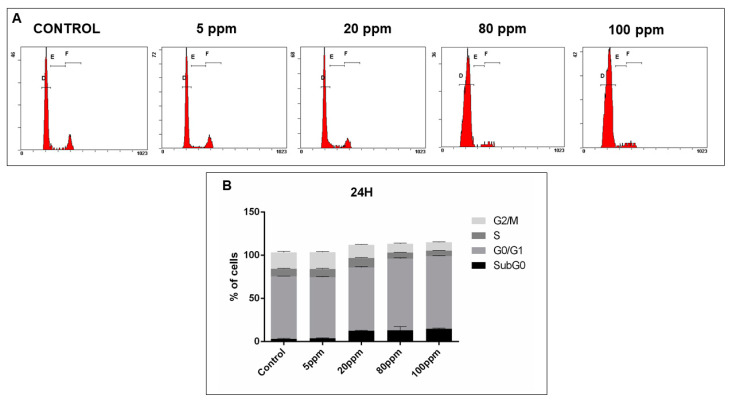
Cell cycle analysis, of LNCaP cells treated with different concentrations of AgNPs for 24 h, assessed using a PI stain and flow cytometry. (**A**)—Representative DNA histograms. (**B**)—Quantitative analysis of PC-3 cells AgNPs treated. Data is expressed as mean ± SEM.

**Figure 11:**
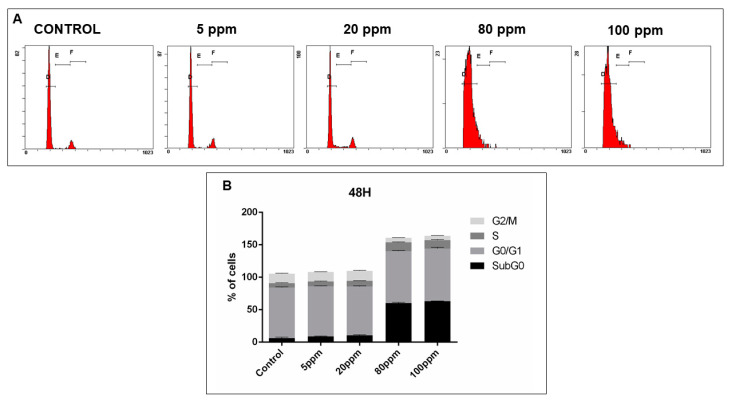
Cell cycle analysis, of LNCaP cells treated with different concentrations of AgNPs for 24 h, assessed using a PI stain and flow cytometry. (**A**)—Representative DNA histograms. (**B**)—Quantitative analysis of PC-3 cells AgNPs treated. Data is expressed as mean ± SEM.

**Figure 12:**
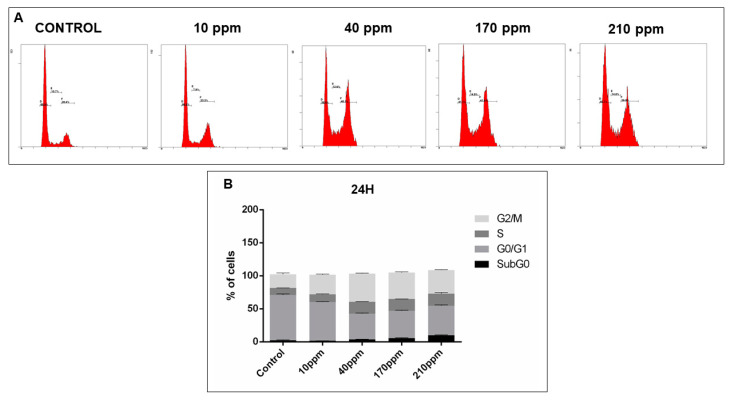
Cell cycle analysis, of PC-3 cells treated with different concentrations of AgNPs for 24 h, assessed using a PI stain and flow cytometry. (**A**)—Representative DNA histograms. (**B**)—Quantitative analysis of PC-3 cells AgNPs treated. Data is expressed as mean ± SEM.

**Figure 13:**
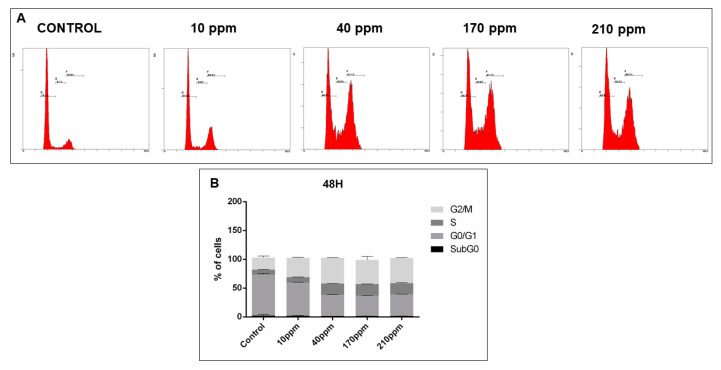
Cell cycle analysis, of PC-3 cells treated with different concentrations of AgNPs for 48 h, assessed using a PI stain and flow cytometry. (**A**)—Representative DNA histograms images. (**B**)—Quantitative analysis of PC-3 cells AgNPs treated. Data is expressed as mean ± SEM.

In order to analyze cell cycle disruptions following AgNPs treatment, PI staining analysis by flow cytometry was used (Figure 10A, Figure 11A, Figure 12A and Figure 13A). 

Regarding LNCaP cells, after 24 h of treatment with AgNPs in concentrations of 20 ppm, 80 ppm and 100 ppm, a significant increase of the number of cells in G0/G1 phase was observed (72.30% ± 0.18 without treatment vs. 73.45 ± 0.53; 83.11 ± 0.59; 84.09 ± 0.39, respectively). Moreover, a subsequent decrease of cells in the G2/M phase was also observed for the same concentrations (19.10% ± 0.64 without treatment vs. 15.20 ± 0.37; 10.29 ± 0.39; 9.92 ± 0.24, respectively). Additionally, there was an increase of the number of cells in subG0 phase (3.20% ± 0.42 without treatment vs. 12.52 ± 0.35; 13.07 ± 2.49; 14.97 ± 0.32, respectively) (Figure 10B).

After 48 h of treatment in concentrations 80 ppm and 100 ppm, a significant increase of the number of cells in G0/G1 phase was observed (78.03 ± 0.53 without treatment vs. 80.13 ± 0.54; 81.12 ± 1.12, respectively). Moreover, a subsequent decrease of cells in the G2/M phase was also observed for the same concentrations (14.61 ± 0.41 without treatment vs. 7.11 ± 0.12; 6.95 ± 0.13, respectively). Additionally, there was an increase of the number of cells in subG0 phase (6.19 ± 0.88 without treatment vs. 60.13 ± 0.78; 63.15 ± 0.15, respectively) (Figure 11B).

However, in PC-3 cells, after 24 h of treatment with AgNPs in concentrations of 40 ppm, 170 ppm and 210 ppm, a significant decrease of the number of cells in G0/G1 phase was observed (68.53% ± 1.05 without treatment vs. 38.83 ± 0.58; 41.30 ± 0.55; 44.70 ± 0.70, respectively). Moreover, a subsequent increase of cells in the G2/M phase was also observed for the same concentrations (20.80% ± 1.19 without treatment vs. 42.80 ± 0.40; 40.03 ± 0.52; 35.87 ± 0.27, respectively) (Figure 12B). 

The same can be observed after 48 h of treatment in concentrations of 40 ppm, 170 ppm and 210 ppm, for both the decrease of the number of cells in G0/G1 phase (70.77% ± 1.28 without treatment vs. 36.67 ± 0.12; 35.50 ± 0.17; 37.77 ± 0.18, respectively) and the increase of cells in G2/M phase (21.03% ± 1.52 without treatment vs. 44.77 ± 0.17; 41.93 ± 3.42; 43.57 ± 0.44, respectively) (Figure 13B). 

### 3.7. AgNPs’ Cellular Uptake

**Figure 14:**
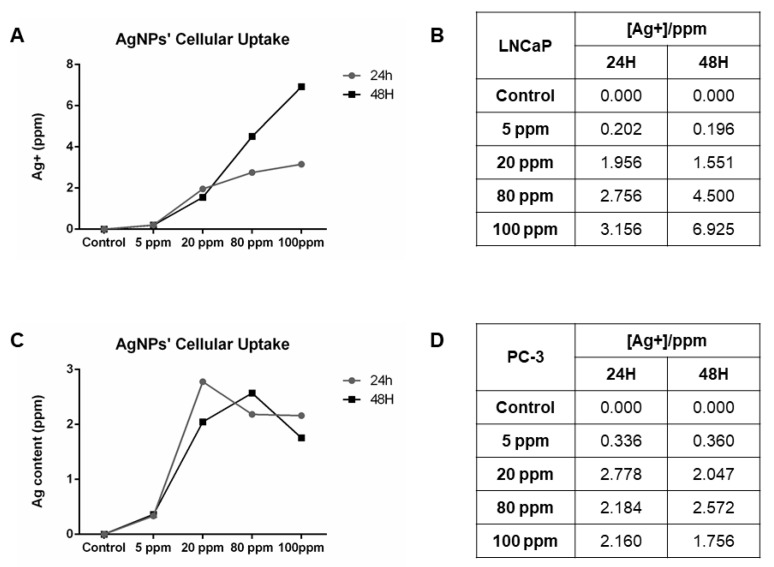
AgNPs’ cellular uptake quantification through Ag^+^ quantification by atomic absorption spectrometry (AAS) in LNCaP cells (**A**,**B**) and in PC-3 cells (**C**,**D**).

In order to analyze the AgNPs’ uptake by LNCaP and PC-3 the Ag^+^ concentration present in cells after 24 and 48 h of AgNPs administration was quantified (Figure 14). Regarding the LNCaP cells, there is an increase in the uptake with the increased concentrations, for both 24 and 48 h of exposure (Figure 14A,B). However, regarding PC-3 cells, a peak of uptake is observed at 20 ppm for 24 h and 80 ppm for 48 h after which the uptake decreases (Figure 14C,D).

## 4. Discussion

In the last few years, there has been a wide scientific interest in the AgNPs’ potential as anticancer agents, more specifically in the mechanisms behind their damage induction in cancer cells [20]. In PC, AgNPs synthesized from several cappings showed cytotoxic power against both LNCaP and PC-3 cell lines suggesting their potential in PC management [21,22,23,24,25,26]. However, to date, no studies with potato starch capped AgNPs have been reported in PC. Moreover, Satapathy and coworkers have studied starch-capped AgNPs’ effect in colon cancer and showed that these AgNPs have less agglomeration when compared with other carbohydrate cappings [15]. A glucose-based capping can pose as an advantage because of cancer cells’ increased energetic demands and consequent increased glucose consumption. In fact, the prostate tissue’s metabolic activity is peculiar due to its organ’s function. In normal conditions, there is an inhibition of Krebs cycle due to the need for citrate which enters the composition of the seminal liquid [27]. However, as PC progresses and because of their increased energetic demands, there is a reactivation of Krebs cycle and, later, a metabolic switch to Warburg effect [28,29,30]. 

In this study, we successfully synthesized starch capped AgNPs with a spherical shape and a medium size of 3 nm-characteristics that favor their uptake and cytotoxicity induction capability since smaller nanoparticles are uptaken more than larger ones and, consequently, will interact more with the various biomolecules inside the cell [10,31]. Moreover, AgNPs with a spherical shape are the most common and there is evidence that this shape, in nanoparticles smaller than 100 nm, is the most favorable to the cellular uptake of the nanoparticle [32].

The green synthesized AgNPs were tested for their anticancer activity against both LNCaP and PC-3 cell lines, using several cytotoxicity, proliferation and morphological assays that evaluate cytoplasmic membrane, mitochondrial and DNA alterations. Starch-capped AgNPs’ cytotoxic capacity had already been studied by Satapathy and co-workers, who investigated their anticancer potential against HCT116 colon cancer cells. They showed that these AgNPs were able to decrease the growth and viability of HCT116 cells in a dose- and time-dependent manner, and that starch alone did not cause any significant growth inhibition in these cells at any concentration. Moreover, they described a decrease of G1 and consequent increase of S phase population, after treatment with starch-capped AgNPs, which is in accordance with what was observed in the present study for the PC-3 cell line [15]. In this study, the loss of normal cell morphology was observed in both cell lines treated with AgNPs after 24 h of exposure. The most recognizable morphological changes were the cell shrinkage and rounded morphology, with production of numerous irregular clusters and floating cells in contrast with the high confluence of monolayer cells observed in the untreated cell, which is consistent with previous studies showing that AgNPs cause morphological changes in a variety of cancer cells [16,33]. 

Starch-capped AgNPs successfully induced cell death in concentrations of 20 ppm and higher. Since no loss of viability was observed when cells were incubated with starch alone, we can assume that the toxicity observed is due to the AgNPs. Interestingly we observed a slight increase of viability of PC-3 cells treated with AgNPs in concentrations of 170 ppm and 210 ppm and at 48 h. This increase of viability may be explained by the decrease of AgNPs’ uptake for these concentrations at 48 h observed in the cells’ silver content measurement performed. It is also important to notice that LNCaP cells showed a consistent increase of uptake which can be responsible for the higher effect of AgNPs in these cells. 

Several studies report that AgNPs exert their toxicity inside the cell in three main locations: Cytoplasmic membrane, mitochondria and nucleus [9]. In our study we observed that AgNPs induced cell death through cytoplasmic membrane disruption. In fact, several authors have reported the presence of membrane blebbing and an increase of the leakage of the cytosolic enzyme Lactate Dehydrogenase from cells treated with different AgNPs (common indicators of cytoplasmic membrane disruption) [34,35]. 

We also observed that these AgNPs reduced the metabolic capacity of cancer cells, indicating possible mitochondria damage. The main scientific evidence regarding mitochondria damage induced by AgNPs shows that this is caused by an increase in ROS production [9,36]. This increase may be due to nanoparticle surface chemistry, depletion of antioxidant molecules (such as glutathione) via binding Ag^+^ to thiol groups, altered enzymatic production of ROS, inhibition of electron transport chain and decrease of superoxide dismutase and catalase’s expression (enzymes that neutralize ROS) [37]. Another possibility, regarding mitochondria’s function impairment is the depolarization of the mitochondrial membrane or the direct inhibition of electron transport chain enzymes [38]. 

Our results show a stop in cellular proliferation of both LNCaP and PC-3 cells treated with AgNPs, which may come as an attempt of the cells to protect themselves from the damage induced by AgNPs. Because the cell cycle is a key regulator of cell proliferation and growth, we performed a cell cycle analysis that confirmed that these AgNPs hampered LNCaP and PC-3 cell cycle. Panzarini and coworkers, have already observed a cell cycle arrest in G2/M in HeLa cells treated with glucose capped AgNPs [39]. Curiously we observed an arrest in G0/G1 in LNCaP cells and an arrest in S and G2/M phases of PC-3 cells treated with AgNPs. The arrest of LNCaP cells has already been observed when treated with 3,6-diazabicyclo [3.3.1] heptane (an acetylcholine nicotinic receptors inhibitor) [40]. Moreover, peperomin E, a natural secolignan, was able to induce G2/M arrest in PC-3 cells, due to an increase of the Bax/Bcl-2 ratio and activation of caspases-3 [41]. The arrest in these different phases of the cell cycle may be due to the more aggressive phenotype of PC-3 cells which allows them to progress more in the cell cycle and may help to understand the lower effect of AgNPs in this cell line when compared to LNCaP cells. Nevertheless, their arrest in G2/M phase may indicate the presence of DNA damage in the PC-3 treated cells since this is known to be associated with the G2-M DNA damage checkpoint, which ensures that cells do not initiate mitosis until damaged or incompletely replicated DNA is sufficiently repaired [42]. 

## 5. Conclusions

In conclusion, the starch-capped AgNPs, synthesized through a green method, successfully induced damage in cytoplasmic membrane and mitochondria, leading to cell cycle arrest and consequent blockage of cell proliferation and death in both LNCaP and PC-3 cells. In the future, it would be interesting to further characterize their composition and stability. Moreover, to better understand their toxicity mechanisms, studies that directly evaluate ROS production and mitochondrial membrane polarization would be useful. These AgNPs cytotoxic potential could also be tested in concomitance with hormone therapy in LNCaP cells in an attempt to delay the hormone resistance acquisition and in PC-3 cells to revert the resistant phenotype. Thus, this data shows the potential of these AgNPs as anticancer agents for different stages of PC disease.

## Data Availability

Data available on request due to restrictions regarding funding policies.

## References

[1] Bray F., Ferlay J., Soerjomataram I., Siegel R.L., Torre L.A., Jemal A. (2018). Global cancer statistics 2018: GLOBOCAN estimates of incidence and mortality worldwide for 36 cancers in 185 countries. CA Cancer J. Clin..

[2] Rawla P. (2019). Epidemiology of Prostate Cancer. World J. Oncol..

[3] Tucci M., Zichi C., Buttigliero C., Vignani F., Scagliotti G.V., Di Maio M. (2018). Enzalutamide-resistant castration-resistant prostate cancer: Challenges and solutions. OncoTargets Ther..

[4] Mottet N., Bellmunt J., Bolla M., Briers E., Cumberbatch M.G., De Santis M., Fossati N., Gross T., Henry A.M., Joniau S. (2017). EAU-ESTRO-SIOG Guidelines on Prostate Cancer. Part 1: Screening, Diagnosis, and Local Treatment with Curative Intent. Eur. Urol..

[5] Davey R.A., Grossmann M. (2016). Androgen Receptor Structure, Function and Biology: From Bench to Bedside. Clin. Biochem. Rev..

[6] Maughan B.L., Antonarakis E.S. (2015). Androgen pathway resistance in prostate cancer and therapeutic implications. Expert Opin. Pharmacother..

[7] Buttigliero C., Tucci M., Bertaglia V., Vignani F., Bironzo P., Di Maio M., Scagliotti G.V. (2015). Understanding and overcoming the mechanisms of primary and acquired resistance to abiraterone and enzalutamide in castration resistant prostate cancer. Cancer Treat. Rev..

[8] Zhang X.F., Liu Z.G., Shen W., Gurunathan S. (2016). Silver Nanoparticles: Synthesis, Characterization, Properties, Applications, and Therapeutic Approaches. Int. J. Mol. Sci..

[9] Khan S.U., Saleh T.A., Wahab A., Khan M.H., Khan D., Khan W.U., Rahim A., Kamal S., Khan F.U., Fahad S. (2018). Nanosilver: New ageless and versatile biomedical therapeutic scaffold. Int. J. Nanomed..

[10] He Y., Du Z., Ma S., Liu Y., Li D., Huang H., Jiang S., Cheng S., Wu W., Zhang K. (2016). Effects of green-synthesized silver nanoparticles on lung cancer cells in vitro and grown as xenograft tumors in vivo. Int. J. Nanomed..

[11] Kummara S., Patil M.B., Uriah T. (2016). Synthesis, characterization, biocompatible and anticancer activity of green and chemically synthesized silver nanoparticles—A comparative study. Biomed. Pharmacother. Biomed. Pharmacother..

[12] Deepak P., Amutha V., Kamaraj C., Balasubramani G., Aiswarya D., Perumal P., Shukla A.K., Iravani S. (2019). Chapter 15—Chemical and Green Synthesis of Nanoparticles and their Efficacy on Cancer Cells. Green Synthesis, Characterization and Applications of Nanoparticles.

[13] Morais M., Teixeira A.L., Dias F., Machado V., Medeiros R., Prior J.A.V. (2020). Cytotoxic Effect of Silver Nanoparticles Synthesized by Green Methods in Cancer. J. Med. Chem..

[14] Cutruzzolà F., Giardina G., Marani M., Macone A., Paiardini A., Rinaldo S., Paone A. (2017). Glucose Metabolism in the Progression of Prostate Cancer. Front. Physiol..

[15] Satapathy S.R., Mohapatra P., Preet R., Das D., Sarkar B., Choudhuri T., Wyatt M.D., Kundu C.N. (2013). Silver-based nanoparticles induce apoptosis in human colon cancer cells mediated through p53. Nanomedicine.

[16] Yuan Y.-G., Zhang S., Hwang J.-Y., Kong I.-K. (2018). Silver Nanoparticles Potentiates Cytotoxicity and Apoptotic Potential of Camptothecin in Human Cervical Cancer Cells. Oxidative Med. Cell. Longev..

[17] Banerjee P.P., Bandyopadhyay A., Harsha S.N., Policegoudra R.S., Bhattacharya S., Karak N., Chattopadhyay A. (2017). Mentha arvensis (Linn.)-mediated green silver nanoparticles trigger caspase 9-dependent cell death in MCF7 and MDA-MB-231 cells. Breast Cancer.

[18] Strober W. (2015). Trypan Blue Exclusion Test of Cell Viability. Curr. Protoc. Immunol..

[19] Kumar S.V., Bafana A.P., Pawar P., Rahman A., Dahoumane S.A., Jeffryes C.S. (2018). High conversion synthesis of <10 nm starch-stabilized silver nanoparticles using microwave technology. Sci. Rep..

[20] Fan W., Yung B., Huang P., Chen X. (2017). Nanotechnology for Multimodal Synergistic Cancer Therapy. Chem. Rev..

[21] He Y., Li X., Wang J., Yang Q., Yao B., Zhao Y., Zhao A., Sun W., Zhang Q. (2017). Synthesis, characterization and evaluation cytotoxic activity of silver nanoparticles synthesized by Chinese herbal Cornus officinalis via environment friendly approach. Environ. Toxicol. Pharmacol..

[22] He Y., Du Z., Ma S., Cheng S., Jiang S., Liu Y., Li D., Huang H., Zhang K., Zheng X. (2016). Biosynthesis, Antibacterial Activity and Anticancer Effects Against Prostate Cancer (PC-3) Cells of Silver Nanoparticles Using Dimocarpus Longan Lour. Peel Extract. Nanoscale Res. Lett..

[23] Chen Y., Yang T., Chen S., Qi S., Zhang Z., Xu Y. (2020). Silver nanoparticles regulate autophagy through lysosome injury and cell hypoxia in prostate cancer cells. J. Biochem. Mol. Toxicol..

[24] Kumari R., Saini A.K., Kumar A., Saini R.V. (2020). Apoptosis induction in lung and prostate cancer cells through silver nanoparticles synthesized from Pinus roxburghii bioactive fraction. JBIC J. Biol. Inorg. Chem..

[25] Firdhouse M.J., Lalitha P. (2013). Biosynthesis of silver nanoparticles using the extract of Alternanthera sessilis—Antiproliferative effect against prostate cancer cells. Cancer Nanotechnol..

[26] Zhang K., Liu X., Samuel Ravi S.O., Ramachandran A., Aziz Ibrahim I.A., Nassir A.M., Yao J. (2019). Synthesis of silver nanoparticles (AgNPs) from leaf extract of Salvia miltiorrhiza and its anticancer potential in human prostate cancer LNCaP cell lines. Artif. Cells Nanomed. Biotechnol..

[27] Eidelman E., Twum-Ampofo J., Ansari J., Siddiqui M.M. (2017). The Metabolic Phenotype of Prostate Cancer. Front. Oncol..

[28] Gonzalez-Menendez P., Hevia D., Mayo J.C., Sainz R.M. (2018). The dark side of glucose transporters in prostate cancer: Are they a new feature to characterize carcinomas?. Int. J. Cancer.

[29] Gonzalez-Menendez P., Hevia D., Alonso-Arias R., Alvarez-Artime A., Rodriguez-Garcia A., Kinet S., Gonzalez-Pola I., Taylor N., Mayo J.C., Sainz R.M. (2018). GLUT1 protects prostate cancer cells from glucose deprivation-induced oxidative stress. Redox Biol..

[30] Morais M., Dias F., Prior J.A.V., Teixeira A.L., Medeiros R. (2021). The Impact of Oxidoreductases-Related MicroRNAs in Glucose Metabolism of Renal Cell Carcinoma and Prostate Cancer. Oxidoreductase.

[31] Cameron S.J., Hosseinian F., Willmore W.G. (2018). A Current Overview of the Biological and Cellular Effects of Nanosilver. Int. J. Mol. Sci..

[32] Albanese A., Tang P.S., Chan W.C. (2012). The effect of nanoparticle size, shape, and surface chemistry on biological systems. Annu. Rev. Biomed. Eng..

[33] Yuan Y.G., Wang Y.H., Xing H.H., Gurunathan S. (2017). Quercetin-mediated synthesis of graphene oxide-silver nanoparticle nanocomposites: A suitable alternative nanotherapy for neuroblastoma. Int. J. Nanomed..

[34] Basta A.H., El-Saied H., Hasanin M.S., El-Deftar M.M. (2018). Green carboxymethyl cellulose-silver complex versus cellulose origins in biological activity applications. Int. J. Biol. Macromol..

[35] Saratale R.G., Benelli G., Kumar G., Kim D.S., Saratale G.D. (2018). Bio-fabrication of silver nanoparticles using the leaf extract of an ancient herbal medicine, dandelion (Taraxacum officinale), evaluation of their antioxidant, anticancer potential, and antimicrobial activity against phytopathogens. Environ. Sci. Pollut. Res. Int..

[36] Nayak D., Kumari M., Rajachandar S., Ashe S., Thathapudi N.C., Nayak B. (2016). Biofilm Impeding AgNPs Target Skin Carcinoma by Inducing Mitochondrial Membrane Depolarization Mediated through ROS Production. ACS Appl. Mater. Interfaces.

[37] Maurer L.L., Meyer J.N. (2016). A systematic review of evidence for silver nanoparticle-induced mitochondrial toxicity. Environ. Sci. Nano.

[38] Al-Sheddi E.S., Farshori N.N., Al-Oqail M.M., Al-Massarani S.M., Saquib Q., Wahab R., Musarrat J., Al-Khedhairy A.A., Siddiqui M.A. (2018). Anticancer Potential of Green Synthesized Silver Nanoparticles Using Extract of Nepeta deflersiana against Human Cervical Cancer Cells (HeLA). Bioinorg. Chem. Appl..

[39] Panzarini E., Mariano S., Vergallo C., Carata E., Fimia G.M., Mura F., Rossi M., Vergaro V., Ciccarella G., Corazzari M. (2017). Glucose capped silver nanoparticles induce cell cycle arrest in HeLa cells. Toxicol. In Vitro Int. J. Publ. Assoc. BIBRA.

[40] Wei H., Lian W., Wang C. (2020). 3,6-diazabicyclo[3.3.1]heptanes Induces Apoptosis and Arrests Cell Cycle in Prostate Cancer Cells. Med. Sci. Monit. Int. Med. J. Exp. Clin. Res..

[41] Li Y., Pan J., Gou M. (2019). The Anti-Proliferation, Cycle Arrest and Apoptotic Inducing Activity of Peperomin E on Prostate Cancer PC-3 Cell Line. Molecules.

[42] Cuddihy A.R., O’Connell M.J. (2003). Cell-cycle responses to DNA damage in G2. Int. Rev. Cytol..

